# A multi-modal MRI analysis of brain structure and function in relation to *OXT* methylation in maltreated children and adolescents

**DOI:** 10.1038/s41398-021-01714-y

**Published:** 2021-11-18

**Authors:** Shota Nishitani, Takashi X. Fujisawa, Daiki Hiraoka, Kai Makita, Shinichiro Takiguchi, Shoko Hamamura, Akiko Yao, Koji Shimada, Alicia K. Smith, Akemi Tomoda

**Affiliations:** 1 Research Center for Child Mental Development, University of Fukui, Fukui, Japan; 2 Division of Developmental Higher Brain Functions, United Graduate School of Child Development, Osaka University, Kanazawa University, Hamamatsu University School of Medicine, Chiba University, and University of Fukui, Osaka, Japan; 3 Life Science Innovation Center, University of Fukui, Fukui, Japan; 4 Gynecology and Obstetrics, Emory University School of Medicine, Atlanta, GA USA; 5 Japan Society for the Promotion of Science, Tokyo, Japan; 6 Department of Child and Adolescent Psychological Medicine, University of Fukui Hospital, Fukui, Japan; 7 Biomedical Imaging Research Center, University of Fukui, Fukui, Japan

**Keywords:** Epigenetics and plasticity, Psychiatric disorders

## Abstract

Child maltreatment dysregulates the brain’s oxytocinergic system, resulting in dysfunctional attachment patterns. However, how the oxytocinergic system in children who are maltreated (CM) is epigenetically affected remains unknown. We assessed differences in salivary DNA methylation of the gene encoding oxytocin (*OXT*) between CM (*n* = 24) and non-CM (*n* = 31), alongside its impact on brain structures and functions using multi-modal brain imaging (voxel-based morphometry, diffusion tensor imaging, and task and resting-state functional magnetic resonance imaging). We found that CM showed higher promoter methylation than non-CM, and nine CpG sites were observed to be correlated with each other and grouped into one index (*OXT*mi). *OXT*mi was significantly negatively correlated with gray matter volume (GMV) in the left superior parietal lobule (SPL), and with right putamen activation during a rewarding task, but not with white matter structures. Using a random forest regression model, we investigated the sensitive period and type of maltreatment that contributed the most to *OXT*mi in CM, revealing that they were 5–8 years of age and physical abuse (PA), respectively. However, the presence of PA (PA+) was meant to reflect more severe cases, such as prolonged exposure to multiple types of abuse, than the absence of PA. PA+ was associated with significantly greater functional connectivity between the right putamen set as the seed and the left SPL and the left cerebellum exterior. The results suggest that *OXT* promoter hypermethylation may lead to the atypical development of reward and visual association structures and functions, thereby potentially worsening clinical aspects raised by traumatic experiences.

## Introduction

Child abuse and neglect (child maltreatment) have severe consequences on the developing brain, even extending into adulthood [1]. It has been widely reported that adults who were abused growing up develop atypical and vulnerable brain substrates [2–5], resulting in a broad range of psychiatric disorders and higher rates of suicide. The development of neocortical regions, which are essential for regulating social interactions such as interpersonal relationships, undergoes major structural and functional reorganization during adolescence and young adulthood [6]. Therefore, it is crucial to identify a target neurobiological mechanism which can be used to intervene in children who were maltreated (CM) prior to this completion of brain reorganization to improve their subsequent quality of life and break the intergenerational transmission of child maltreatment. However, neurobiological research conducted on child maltreatment has been limited, regardless of the field, because obtaining parental informed consent for underage children remains challenging [7–9]. Nonetheless, we assessed the neurobiological impact of child maltreatment using cognitive and clinical assessments [10–12] and brain imaging [13–16] under the care of appropriate institutions.

DNA methylation, one of the epigenetic modifications, mediates the interrelationship between external environmental factors and long-lasting phenotypic changes. However, the neurobiological impact of DNA methylation alterations triggered by prolonged maltreatment exposures on social brain development in children remains unclear. Dysregulation of the oxytocinergic system plays an essential role in attachment malformation often found clinically in CM [17]; therefore, we previously assessed DNA methylation targeting the promoter region of the oxytocin receptor (*OXTR)* gene based on the hypothesis that child maltreatment epigenetically affects the gene, thereby leading to an atypical vulnerable brain structure. We demonstrated that higher promoter region DNA methylation levels at the specific CpG sites were more prevalent in CM and were associated with reduced gray matter volume (GMV) in the orbitofrontal cortex [18]. Although not in children themselves, associations between *OXTR* methylation and early childhood adversity in adults have also been reported [19–21]. Moreover, no epigenetic studies have yet assessed the link between child maltreatment and DNA methylation in the gene coding for oxytocin (*OXT*), although the oxytocinergic system is driven by the combination of *OXTR* and *OXT*.

There are strong indications that *OXT* methylation is associated with the brain substrates involved in human sociability. Haas et al. provided the first comprehensive experimental evidence that promoter region methylation, which is in a CpG island of the *OXT* gene, in unison rather than independently, was associated with reduced brain activity in the superior temporal gyrus and inferior frontal gyrus in response to two types of empathetic tasks, reduced GMV in the fusiform gyrus which was in the empathetic network, as well as with behavioral task performance on facial expression recognition involved in mentalizing [22]. The mean promoter methylation status also seems to be associated with stressful life events in inpatients suffering from major depression [23]. Recently, we also found that the promoter region, again in unison, correlated with higher scores on personal distress, an aspect of affective empathy, and lower GMV in the right inferior temporal gyrus in mothers [24]. Based on these studies, it is suspected that methylation in this promoter region may occur in unison because of its nature of being within a CpG island and may lead to vulnerability in socially relevant brain substrates.

Here, we examined the relationship between *OXT* methylation, child maltreatment status, and its effects on brain structures and functions as measured by multi-modal magnetic resonance imagining (MRI) in the same dataset as previously reported [18]. First, we acquired epigenetic data from saliva samples and investigated the cross-sectional case-control comparison of *OXT* methylation. Second, we comprehensively analyzed the correlation between the methylation of *OXT* and 1) GMV using voxel-based morphometry (VBM) approach, 2) structural connectivity using diffusion tensor imaging (DTI) approach, and 3) regional brain activation in response to a rewarding task [13]. In addition, we conducted a random forest regression model analysis to determine which periods of exposure and types of maltreatment particularly contributed to *OXT* methylation alterations. We also compared 4) the resting-state functional connectivity (rsFC) between CM subgroups identified using the regression model analysis. Thus, our central hypothesis was that the *OXT* promoter region methylation levels would be modified by the exposures of maltreatment with specific periods or types and would alter brain structures and functions implicated in social interactions via atypical brain development during childhood and adolescence.

## Materials and methods

### Ethics statement

The study protocol was approved by the Ethics Committee of the University of Fukui, Japan (Assurance no. FU23–43 and 20190107), and the study was carried out in accordance with the Declaration of Helsinki and the Ethical Guidelines for Clinical Studies of the Ministry of Health, Labor and Welfare of Japan. All children and a parent or director of a child welfare facility provided written informed assent and consent, respectively.

### Participants

We leveraged the dataset used in our previous study [18]. Briefly, the CM group consisted of 24 children (12.6 ± 2.2 years old) with prolonged maltreatment experiences (ICD-10-CM Code T74) who had been legally removed from the care of their biological parents by Child Protective Service (CPS) and sheltered in residential childcare facilities. The CM had experienced physical, emotional, sexual abuse, and/or neglect early in life prior to coming into care. The control group consisted of 31 children (14.9 ± 2.2 years) with no history of maltreatment (non-CM).

### Saliva collection and DNA extraction

Saliva samples were acquired using the Oragene Discover OGR-500 kit (DNA Genotek Inc. Ottawa, Canada). DNA was extracted using prepIT®•L2P reagent (DNA Genotek) and quantified as described previously [18, 25].

### *OXT* methylation (microarray)

**Fig. 1:**
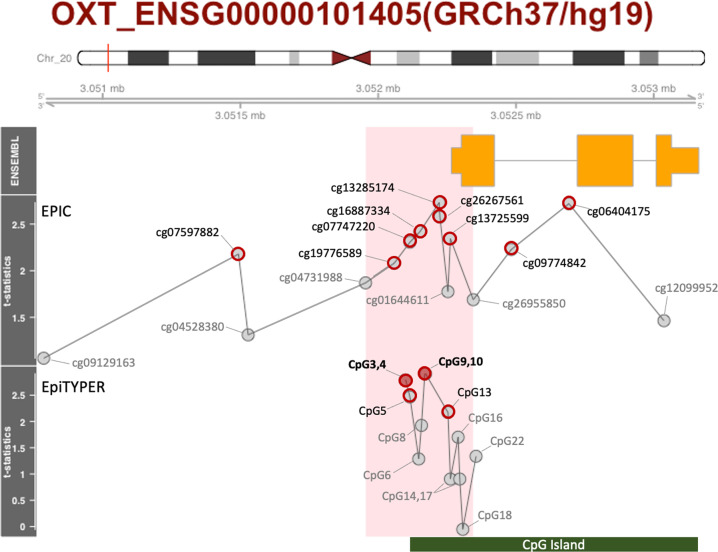
Localization and structure of the *OXT* gene on chromosome 20 (GRCh37/hg19) and the location of the CpG probes from EPIC (upper) and CpG fragments from EpiTYPER (lower). The *t* statistics represent the results of multiple regression analyses between each CpG and child maltreatment. Closed and open red circles represent FDR < 0.05, and FDR < 0.10, respectively. The light pink shaded area represents the clustered region defined by a factor analysis.

Genomic DNA (500 ng) was processed using the Illumina® MethylationEPIC array. The BeadChips were scanned using iSCAN (Illumina Inc., San Diego, CA, USA), and the methylation level (*β* value) was calculated for each queried CpG locus using the GenomeStudio Methylation Module software (Illumina Inc., San Diego, CA, USA), followed by a Psychiatric Genomics Consortium-Epigenome-Wide Association Studies quality control pipeline [26]. Probes containing single nucleotide polymorphisms (SNPs; based on 1000 Genomes) within ten base pairs of the target CpG were maintained. Normalization of probe distribution and background differences between Type I and Type II probes was conducted using beta mixture quantile normalization (BMIQ) after background correction. Following normalization, batch effect removal as implemented in the ComBat procedure of the SVA package in Bioconductor was used to account for sources of technical variations, including batch and positional effects, which can cause spurious associations. Consequently, 794,120 probes were used for further analysis. Saliva contains a heterogeneous mixture of cell types that differ in proportions. Using the EpiDISH method [27], we estimated the proportion of epithelial cells derived from salivary DNA and entered it as a covariate in our statistical models. From this dataset, we evaluated 15 *OXT* CpG probes (Fig. 1, Supplementary Material).

### *OXT* methylation (targeted)

One microgram of DNA was treated with bisulfite using the EpiTect Bisulfite Kit (Qiagen, Hilden, Germany). DNA methylation of CpG sites in the promoter region of the *OXT* gene (chr20: 3,052,266–3,053,162; hg19 build) was analyzed using EpiTYPER (MassARRAY system; Agena Biosciences., San Diego, CA, USA) according to the manufacturer’s instructions. Forward (aggaagagagTTTTTTTGTTTTATTTTAGTGGTTTAGG) and reverse (cagtaatacgactcactatagggagaaggctTCTTACCTCCCAAAAAACAATTCTA) primers corresponding to chr20:3,052,009–3,052,392 were designed using EpiDesigner (Agena Bioscience, San Diego, CA, USA), and the spectrum characteristics were validated with RSeqMeth [28]. Further details were described in a previous study [22] where SN and AKS contributed as co-authors.

### Maximum likelihood factor analysis (MLFA)

We conducted an MLFA to develop an *OXT* methylation index (*OXT*mi) that reflects the characteristics of the representative probes in the promoter region [29].

### Psychological assessments

We assessed clinical aspects under the supervision of pediatric psychology clinicians (ST and AT). The Child Abuse and Trauma Scale (CATS) was administered to assess trauma and maltreatment in early childhood [30]. The Depression Self-Rating Scale for Children (DSRSC) was used to measure depressive symptoms [31]. Behavioral and emotional problems were assessed using the Strengths and Difficulties Questionnaire (SDQ) (Goodman [54]) and the Child Behavior Checklist (CBCL) [32]. To assess the attachment style, the Japanese version of the Attachment Style Measures revised to the multiple-point Likert-type scale (Internal Working Model Scale; IWMS) [33–35] was used, with confirmed reliability and validity of both the original [33, 34] and the Japanese version [35, 36].

### Brain image acquisition and pre-processing

Image acquisition was performed using a 3 T scanner (Discovery MR 750; General Electric Medical Systems, Milwaukee, WI, USA) with a 32-channel head coil. Functional images using monetary reward tasks and those in a resting-state were acquired with a T2*-weighted gradient-echo echoplanar imaging (EPI) sequence. High-resolution images were acquired by a 3D T1-weighted fast spoiled gradient recalled imaging sequence. The DTI acquisition consisted of a single-shot EPI pulse sequence. T2*-weighted functional and T1-weighted structural images were pre-processed using Statistical Parametric Mapping (SPM) 12 (Wellcome Trust Center for Neuroimaging, London, UK) and the toolboxes implemented on MATLAB R2016b (MathWorks, Natick, MA, USA). The DTI data were pre-processed using the Oxford Center for Functional MRI of the Brain (FMRIB) Software Library (FSL; http://www.fmrib.ox.ac.uk/fsl). More details on the acquisition parameters and pre-processing of each imaging sequence are provided in the Supplementary Methods.

### Monetary reward task for fMRI

A subset of 33 participants (CM: 14, non-CM: 19) performed a block-design gambling task used in our previous study [13]. Briefly, participants were asked to choose one of the three cards by pressing a button. Each card was randomly assigned to Japanese Yen (JPY) 0, 30, or 60 (100 JPY is equivalent to 1 USDollar). Three conditions of eight trials were performed (24 s). Further details for the monetary reward task were described in Supplementary Methods.

### Maltreatment history

The presence of a potential “sensitive period,” during which exposure to child maltreatment may be more strongly associated with *OXT* methylation, was assessed using random forest regression with conditional inference trees (“cforest” in R package party) [37], as we have used in previous studies [5, 38]. Two separate analyses were conducted to evaluate the importance of potential predictors of *OXT* methylation alterations. In the first analysis, we determined the importance of exposure during specific time periods, including in utero exposure to mothers’ experiencing domestic violence and direct exposure to neglect or physical, emotional, or sexual abuse from birth to 18 years of age, annually. Years were scored either zero or one for exposure to any type of maltreatment during the year. In the second analysis, a random forest regression was carried out to determine the comparative importance of exposure to neglect versus physical, emotional, or sexual abuse. The overlapping number of types of maltreatment was included as an additional predictor because the multiplicity of exposure may represent a more important determinant than any specific type of exposure. Each forest consisted of 200 trees, with four variables randomly selected for evaluation at each node.

### Statistical analysis

To assess the association between *OXT* methylation at 15 CpG probes (EPIC) or 10 fragments (EpiTYPER) and child maltreatment, multiple regression analyses were performed using CpGassoc (Barfield et al. [53]). In these analyses, DNA methylation at each CpG probe or fragment was entered as a dependent variable, and each group (CM or non-CM) was entered as an independent variable. Age, sex, full-scale intelligence quotient (FSIQ) on the Wechsler Intelligence Scale for Children-Fourth Edition or the Wechsler Adult Intelligence Scale-Third Edition, and the proportion of epithelial cells were entered as covariates, and the results were thresholded at Benjamini–Hochberg (BH) *FDR* < 0.05. After defining *OXT*mi by MLFA, we conducted multiple regression analyses using the same model, except for replacing the methylation dataset with *OXT*mi to confirm that *OXT*mi represents the initial analysis results. The regression validation was conducted by using R code in Supplementary Material. We also examined multiple regression analyses between *OXT*mi as a dependent variable and psychological assessments as independent variables instead of the group.

To test for the association between *OXT*mi and functional structural imaging, multiple regression analyses were performed using SPM12 (https://www.fil.ion.ucl.ac.uk/spm). For functional images using monetary reward tasks, individual task-related activation was evaluated at the first level. Three regressors for each condition (i.e., HMR, LMR, and NMR) were modeled at the onset of each block (duration of 24 s), which were convolved with a canonical hemodynamic response function to obtain the expected task-related signal change. The weighted sum of the parameters estimated during the individual analyses consisted of “contrast” images. Global signal changes were utilized to remove confounding factors such as scanner gain. At the second level, contrast images corresponding to each condition for each participant were used for group analyses with a random-effects model to obtain population inferences. One-sample *t*-tests were used to depict the relevant brain regions for *OXT*mi. To exclude the effects of age, sex, and FSIQ, we included these measures as covariates. Significant signal changes for each contrast were assessed using *t*-statistics on a voxel-by-voxel basis. Once we identified the brain regions, we compared the *β* values between the groups by *t*-test.

For resting-state fMRI, a seed-to-voxel analysis was conducted using the CONN toolbox. At the first level of analysis, the parameters of realignment and scrubbing obtained by the pre-processing process were entered as covariates. At the second level, we conducted a seed-to-voxel analysis in which physical abuse experience, age, sex, and FSIQ were included as covariates to examine the difference in functional connectivity with and without physical abuse experience. Seeds were set based on the results of the fMRI analysis.

For structural images, we performed a multiple regression analysis using VBM on SPM12. Age, sex, FSIQ, and total GMV were included as covariates in the model. The resulting set of voxel values used for comparison generated a statistical parametric map of the *t*-statistic, SPM[*t*], which was transformed to a unit normal distribution (SPM[*Z*]). Significant clusters were localized using the Neuromorphometrics atlas (Neuromorphometric, Inc.; http://www.neuromorphometrics.com/). To control for multiple statistical testing for the entire brain volume, we maintained a cluster-level corrected *FDR* at *P* < 0.05, using a conservative voxel-level threshold of *P* < 0.001.

For DTI data, we utilized tract-based spatial statistics (TBSS), a voxel-wise approach, to examine the fiber tracts of the whole brain. Compared to the traditional region-of-interest approach, the TBSS method allows better sensitivity, objectivity, and interpretability of DTI analyses [39]. Further analysis details were shown in Supplementary Methods.

### Meta-analytic decoding of network function using NeuroSynth

The functional properties of *OXT*mi-related structural regions were decoded using a large-scale database-informed meta-analytic approach as implemented in NeuroSynth [40]. A meta-analytic map associated with the identified region coordinates was derived. Further, the terms (excluding terms for brain regions) and ranked by the *z*-score.

## Results

### *OXT* methylation differences

We revealed that nine EPIC probes showed trends for significant association with child maltreatment across the *OXT* gene (*Ps*_*uncorr*_ < 0.05, *FDRs* < 0.10, Supplementary Table S1 and Fig. 1). Of these, six were serially located in the promoter region. We proceeded to validate the results using targeted analysis as an alternate method. We found that two CpG fragments were significantly and positively associated with child maltreatment (*FDRs* < 0.05, Supplementary Table S2 and Fig. 1). The genomic locations of the three CpG sites (CpG 5, 13, and 14) in EpiTYPER were identical to those of the EPIC probes (cg07747220, cg01644611, and cg13725599), and their methylation was robustly correlated (Supplementary Fig. S1).

### Maximum likelihood factor analysis (MLFA)

MLFA identified one reliable factor (eigenvalue 8.15, loadings 0.606–0.881) that explained 36.6% of the variance (Supplementary Table S3 and Supplementary Fig. S2). The nine probes serially located in the promoter region loading on the primary factor were subsequently averaged to form the *OXT* methylation index (*OXT*mi). They were also clearly correlated with each other (Supplementary Fig. S3). Finally, we confirmed that *OXT*mi was the representative index, proving that it was significantly associated with child maltreatment in the multiple regression model. The linear regression analysis using standard errors, which are robust to heteroskedasticity (see R code in Supplementary Material), revealed that the model was significant (*F* [5, 41] = 13.87, *P* = 1.9E−08) and that child maltreatment was a significant predictor of *OXT*mi (*β* = 0.42, *t* = 2.42, *P* = 0.02), while age, sex, and FSIQ were not (*β* = 0.11, *t* = 0.81, *P* = 0.42; *β* = 0.22, *t* = 1.82, *P* = 0.07; *β* = 0.18, *t* = 1.10, *P* = 0.27, respectively). The proportion of buccal epithelial cells had a significant effect on *OXT*mi (*β* = 0.72, *t* = 8.08, *P* = 1.5E−10); however, this confounding effect was removed by including this item in the model. Hence, we used *OXT*mi for subsequent analyses.

### *OXT*mi and brain structures and functions

**Fig. 2:**
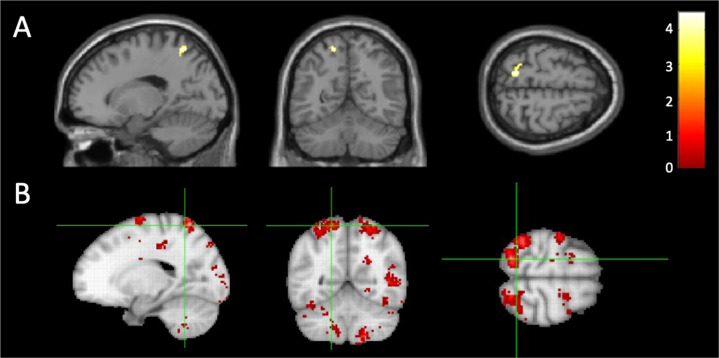
Results of the multiple regression analysis between *OXT*mi and the regional gray matter volume (GMV). **A** Brain region showing negative correlations between the degree of *OXT*mi and GMV in the left SPL (*x* = −17, *y* = −56, *z* = 65 [BA 7], *T* = 4.50, cluster size = 157 voxels) as determined by the multiple regression analysis. The statistical threshold for the contrasts was voxel-level *P* < 0.001 uncorrected for height and cluster-level *P* < 0.05 *FDR*-corrected for multiple comparisons. The color bar denotes the *t*-statistic range. **B** Brain activation map based on a meta-analysis of 118 studies related to the term “action observation,” which was the most significant term associated with the left SPL revealed by “NeuroSynth” [40].

**Fig. 3:**
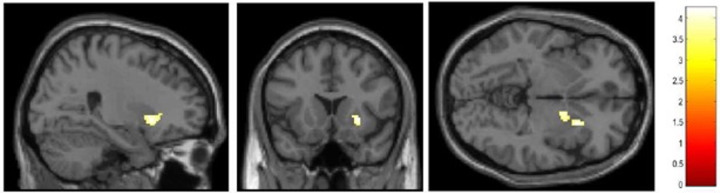
Results of the multiple regression analysis between *OXT*mi and the regional function for monetary reward task. Brain region showing negative correlations between the degree of *OXT*mi and activation in the right putamen (*x* = 26, *y* = 16, *z* = −4, *T* = 4.26, cluster size = 165 voxels) after correcting for age, sex, and FSIQ as determined by multiple regression analysis. The statistical threshold for the contrasts was voxel-level *P* < 0.001 uncorrected for height, corrected to *P* < 0.05 using the cluster size. The color bar represents the *T*-statistic range.

*OXT*mi was negatively correlated with the regional GMV in the left superior parietal lobule (SPL; Fig. 2A) (Broadman area (BA) 7; MNI *x* = −17, *y* = −56, *z* = 65, *T* = 4.50; cluster size = 157 voxels, *P* = 0.047, *FDR*-corrected for cluster level). The functional decoding of the coordinates for the left SPL revealed by NeuroSynth [40] showed terms mostly associated with vision, motor, and their coordination required action mirroring, such as “action observation (*z*-score = 6.1)”, “pointing (*z*-score = 4.2)”, “motor imagery (*z*-score = 4.0)”, and “imitation (*z*-score = 3.8)”. To decode the type of functions the left SPL involved, we showed a brain activation map based on a meta-analysis of 118 studies related to the term “action observation,” which was the most significant term (Fig. 2B). No DTI Scalars (FA, MD, AD, and RD) were significantly correlated with *OXT*mi. *OXT*mi was negatively correlated with the regional reward functions in the right putamen (Fig. 3; MNI coordinates *x* = 26, *y* = 16, *z* = −4, *T* = 4.26; cluster size=165 voxels, *P* < 0.001 uncorrected for peak level, corrected to *P* < 0.05 using the cluster size). There were no significant differences in the GMV of left SPL (*t* = −0.76, *P* = 0.57) and the activation of right putamen (*t* = 0.06, *P* = 0.88) *β* values were observed between groups.

### *OXT*mi and psychological assessments

Trauma and maltreatment assessed by CATS were significantly associated with *OXT*mi in total (*β* = 0.29, *t* = 2.21, *P* = 0.03) and CM (*β* = 0.53, *t* = 2.3, *P* = 0.04) (Table 1). No other type of psychological assessments were significantly associated.

**Table 1:** Multiple linear regression results of *OXT*mi for the association with the clinical symptoms.

*OXT*mi	Total			CM (*n* = 24)			Non-CM (*n* = 31)		
	*β*	Statistics	*P*	*β*	Statistics	*P*	*β*	Statistics	*P*
CATS total^a^	0.29	2.21	0.03*	0.53	2.30	0.04*	−0.27	−1.80	0.09
SDQ total^b^	0.17	1.34	0.19	0.07	0.34	0.74	−0.10	−0.60	0.56
CBCL total^c^	−0.07	−0.49	0.62	−0.16	−0.81	0.43	−0.22	−1.63	0.12
DSRSC^d^	0.13	1.00	0.32	−0.06	−0.29	0.78	0.007	0.04	0.97
*IWMS*^b^									
Secure	−0.09	−0.80	0.43	−0.04	−0.16	0.88	0.08	0.53	0.60
Avoidant	0.08	0.60	0.55	0.06	0.26	0.80	0.06	0.43	0.67
Ambivalent	0.22	1.89	0.06	0.22	1.01	0.32	0.04	0.27	0.79
Insecure	0.18	1.50	0.14	0.17	0.77	0.45	0.06	0.40	0.69

*CATS* Child Abuse and Trauma Scale (Sanders and Becker-Lausen [30]), *SDQ* strengths and difficulties Questionnaire (Goodman [54]), *CBCL* Child Behavior Checklist (Achenbach [32]), *DSRSC* depression self-rating scale for children (Birleson [31]), *IWMS* Internal Working Models Scale (Takuma and Toda [35]).

Bold values identify statistical significance (p < 0.05).

Some subjects’ data were not available:

^a^CATS (CM: *n* = 9, non-CM: *n* = 3).

^b^SDQ and IWMS (CM: *n* = 7).

^c^CBCL total (CM: *n* = 5).

^d^DSRSC (CM: *n* = 6).

*P < 0.05.

### *OXT*mi and maltreatment history

**Fig. 4:**
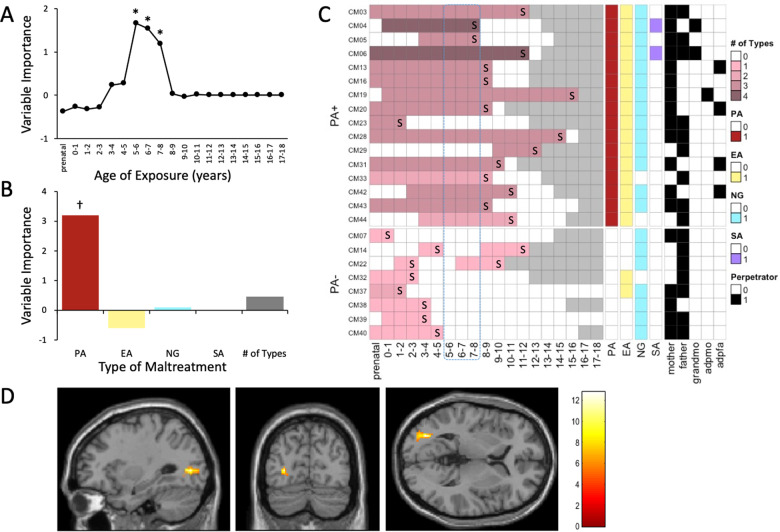
Results of the sensitivity analyses for maltreatment history in CM and their impact on the brain functional connectivities. **A** Maximal sensitivity by age of exposure (regardless of type and number). Results of a random forest regression with conditional trees indicated the importance of exposure to early maltreatment from prenatal to 18 years of age on the *OXT*mi. Importance is indicated by degradation in fit, as suggested by the increase in mean square error (MSE), following effective elimination of each age from the model by permutation. **B** Maximal sensitivity by type and number of maltreatments (regardless of age of exposure). *P < 0.05 (uncorrected). ^†^*P* < 0.10. **C** Structure of maltreatment history for each individual. Gray block: Not to reach the age, S sheltered, PA physical abuse, EA emotional abuse, NG neglect, SA sexual abuse, mother: biological mother, father: biological father, grandmo: grandmother, adpmo: adoptive mother, adpfa adoptive father. **D** PA+ (*n* = 7) vs. PA− (*n* = 4) comparison for resting state fMRI (rs-fMRI). The color bar denotes the *T*-statistic range.

The most important temporal predictor of *OXT*mi consisted of whether or not CM were exposed to maltreatment at 5–8 years of age, with a peak occurring at 5–6 years (*r* = 0.232). The probability of obtaining these three dots (5–6, 6–7, and 7–8 years) of age with this combined degree of importance was significantly low (Fig. 4A, *Ps* < 0.05). The degree of *OXT*mi could also be predicted with reasonable accuracy based on the type of maltreatment (*r* = 0.222). Physical abuse (PA), a specific type of maltreatment, emerged as the most important predictor (Fig. 4B, *P* = 0.07). The clinical aspects and maltreatment history for PA+/− are depicted in Supplementary Table S4 and Fig. 4C. In addition, we found a significant dose-dependent relationship between the subgroups (PA+, PA−, and non-CM) and *OXT*mi (*r*_*part*_ = 0.32, *P* = 0.02, Supplementary Fig. S4). A significant main effect of PA+ (*n* = 7) compared to PA− (*n* = 4) in increased rsFC was found between the right putamen and left SPL (Fig. 4D; BA 7; MNI coordinates *x* = −22, *y* = −74, *z* = 44, *T* = 11.11; cluster size = 128 voxels) and left cerebellum exterior (MNI coordinates *x* = −20, *y* = −52, *z* = −56, *T* = 13.10; cluster size = 125 voxels). No between-group differences in rsFC were observed in the seeds of the left SPL.

## Discussion

We revealed that CM had elevated methylation in the *OXT* gene promoter region. This series of methylations were correlated with each other and clustered into an *OXT*mi based on factor analysis. We assessed the relationship between *OXT*mi and brain structures and functions using multi-modal MRI and found that the greater the *OXT*mi, the smaller the left SPL GMV. However, there was no correlation with white matter structures. Additionally, we found that the greater the *OXT*mi, the lower the activity of the right putamen during the reward task. We then examined the relationship between *OXT*mi and psychological assessments and found that a greater *OXT*mi was associated with stronger trauma and maltreatment. Finally, we investigated the relationship between *OXT*mi and maltreatment history and found that hypermethylation of *OXT*mi was associated with PA and maltreatment exposure during 5–8 years of age. We compared PA+ and PA− and found that PA+ had higher trauma and maltreatment and increased functional connectivity between the right putamen and left SPL and cerebellum. These results indicate that hypermethylation of the *OXT* gene promoter region may be associated with exposure to severe abuse, including PA, which persists beyond the age of 5 years and leading to atypical development of reward and visual association functions.

Hypermethylation in the promoter region of the *OXT* gene, found in this study to be associated with child maltreatment, was in the same region and coincided with the direction of the effect in previous studies that examined the association between the intermediate phenotype of empathy and depression [22–24]. However, no study has focused on *OXT* promoter methylation and examined its relationship with child maltreatment, and the present study is the first to examine it. Our factor analysis extracted a series of nine consecutive methylations lasting 391 bp (Chr20: 3051954–3052345, GRCh37/hg19) as a single factor without facing the issue of extracting factors constructed of disparate sites while ignoring genomic geographic information.

Using *OXT*mi, whole-brain VBM analysis revealed an association with left SPL GMV reduction. The SPL is included in the dorsal attentional network, and together with eye movement coordinated with the function of the frontal eye field, it regulates selective attention, spatial cognitive functions, and motor imaginary [42]. In adults with Asperger syndrome, treatment with oxytocin (OT) nasal spray showed enhanced brain activity, including in the left SPL, during a facial expression recognition task compared to the placebo condition [43]. This implies that the left SPL is the target region for OT. Indeed, OT is considerably expressed in the parietal cortex, as represented in the RNAseq dataset of postmortem developing brains (Supplementary Fig. S5). A meta-analysis in adults revealed that the left SPL showed decreased activity toward socio-affective cues with higher scores on the childhood trauma questionnaire [44]. Additionally, a recent brain imaging study in response to gaze in children and adolescents with a disruptive behavior disorder with psychopathic traits, which are often found in CM due to disrupted secure attachment formation [45–48], reported reduced activity in the dorsal attentional network, including the left SPL, in response to eye movements of fearful faces [49]. Although we have previously reported structural atypicality in the visual cortex in young adults [2, 5] and children [14], there have been no reports of abnormalities in visual or its associated functions in CM. Given the hypermethylation of the *OXT* gene promoter, which was associated with reduced left SPL GMV in the dorsal attention network, and the structural atypicality of the visual cortex, it is likely that there is a certain degree of atypicality in CM in the coordination of gaze recognition of others and their own eye movements, which may be driven by OT.

We previously found that CM diagnosed with reactive attachment disorder showed decreased activity in the dorsal striatum, including the right putamen, and may be vulnerable to reward system functioning [13]. In the present study, we found that the activity of the right putamen decreased with higher *OXT*mi. The OT receptor OXTR, however, is mainly localized in the reward system network of the midbrain ventral tegmental area, nucleus accumbens, and orbitofrontal cortex. If methylation of the *OXT* promoter region suppresses its expression and OT synthesis, the amount of OT acting on the receptors in the reward system would also be reduced, which may be involved in weakening the reward system function. We also conducted a randomized controlled trial to examine the effects of nasal OT in CM and found that nasal OT enhanced the activity of the right putamen compared to the placebo [41]. This further supports this idea.

Of the psychological assessments, greater *OXT*mi was linked to a greater number of trauma and maltreatment experiences, but not prosocial behavior, psychopathology, depressive symptoms, or attachment style. The intermediate phenotypic atypicality described thus far, such as decreased left SPL GMV and decreased reward system activity in the right putamen, may be linked to the frequency and extent of various types of trauma and maltreatment experiences. Additionally, analyses that examined the relationship between *OXT*mi and maltreatment history characterized *OXT*mi as being associated with greater susceptibility to exposure during ages 5–8 years and PA+. However, we were unable to extract the exclusive effects of a specific period or PA+ from the effect of overlapping or prolonged maltreatment. Therefore, the characteristics found in the analyses had solid implications for dichotomizing the severity of abuse victimization. On splitting the CM into PA+/−, PA+ revealed multiple types of abuse, while PA− had almost only neglect, as shown in Fig. 4C. Moreover, PA + CM were exposed to maltreatment for long periods after the age of 5 years, while most PA− were sheltered before the age of 5 years and lived safely in institutions after that. Furthermore, the rs-fMRI comparison between PA+ and PA− showed that rsFC with the left SPL and cerebellum was greater in PA+ when the right putamen was set as the seed. As discussed previously, the SPL is included in the dorsal attentional network and regulates selective attention, spatial cognitive functions, and motor imaginary [42], and we found that the left SPL GMV reduction was associated with *OXT*mi hypermethylation in the whole-brain VBM analysis. It may be that prolonged and severe abuse, as in PA+, leads to aversive learning of punishment when a human face enters the visual field, which leads to atypicality, overexcitement of functional connections between the dorsal striatum, including the putamen, and the left SPL.

This study had five major limitations. First, the sample size was relatively small. For example, we found that *OXT*mi, which was associated with CM, was significantly associated with the GMV of left SPL and the putamen activity. However, we could not detect group differences in these brain volumes and activities themselves. Reproducibility in larger datasets is necessary to confirm the results, but obtaining research consent from CM is exceptionally complicated [7–9]. Second, the CM group in the present study was a heterogeneous population in terms of the type of maltreatment they had been experienced, as shown in Fig. 4C. However, different types of maltreatment often coexist in the nature of child maltreatment, and overlaps would be considerable. Therefore, to examine a specific effect of only a single type of maltreatment, a larger population would be needed to make the subset for it. Third, because we were analyzing salivary DNA methylation, our data may not necessarily reflect the state of the brain due to the tissue specificity of methylation patterns [50]. We have tested whether saliva correlates with brain *OXT* methylations using the IMAGE-CpG database [51] (Supplementary Table S5). The EPIC-based results seemed to show almost negative correlations in the region that constructed *OXT*mi, but it did not match with 450K-based results. We still need to know if this probable Caucasian-based database is appropriate for interpreting the results taken from different races since there are a large number of methylation quantitative trait loci across the genome that are influenced by genetic polymorphisms. Forensic autopsy brains of children who died due to abuse should be examined, which is another way to overcome the tissue specificity issue. Fourth, this study did not assess whether methylation of the *OXT* gene promoter region inhibited the expression and reduced the amount of OT released. Finally, this dataset does not contain all the imaging data available because participants were relatively younger for brain MRI, aged from 9-year-old, and some of them could not be successfully imaged due to unacceptable body movements (Supplementary Table S6). There were no non-CM rs-fMRI data.

The present study showed that child maltreatment resulted in hypermethylation of the *OXT* gene promoter, which was associated with decreased left SPL GMV in the dorsal attention network, decreased right putamen function for the reward system. Maltreatment with PA+ or prolonged maltreatment lasting beyond the age of 5 years with greater trauma experiences were associated with this hypermethylation, resulting in atypical FC between the reward system and SPL and cerebellum. Trauma-Focused Cognitive Behavioral Therapy (TF-CBT), and Eye Movement Desensitization and Reprocessing (EMDR), which are currently the standard treatments for post-traumatic stress disorder [52], are means of addressing these issues. The results of this study are considered to have affirmed their effectiveness since trauma symptoms are the core pathology in CM and because there is atypicality in visual association functions such as eye movements and attentional functions. Therefore, if the inhibition of OT synthesis caused by promoter methylation results in adverse symptoms, early intervention and treatment combined with interventional supplementation of oxytocinergic function through OT administration may be more effective at improving clinical aspects raised by traumatic experiences in CM.

## Supplementary information

Supplementary Information

Supplementary Methods

Dataset 1

Dataset 2

Dataset 3

## Data Availability

R code is available as Supplementary Material.
