# Supplementary material for: A multi-modal MRI analysis of brain structure and function in relation to OXT methylation in maltreated children and adolescents
Source: Transl Psychiatry. 2021 Nov 18;11:589. doi: 10.1038/s41398-021-01714-y (PMC8599663; doi:10.1038/s41398-021-01714-y)
Supplement: Supplementary file 1 — Supplementary Information [file 41398_2021_1714_MOESM1_ESM.docx]

**Supplementary Table S1.** Statistical analysis results using multiple regression for each EPIC probe

| CPG.Labels | *T*.statistic | *P*.value | *FDR* (BH) | CHR | MAPINFO | UCSC_RefGene_Group | Relation_to_UCSC_CpG_Island |
| --- | --- | --- | --- | --- | --- | --- | --- |
| cg09129163 | 1.06 | 0.29 | 0.29 | 20 | 3050786 | TSS1500 | N_Shore |
| cg07597882 | 2.18 | 0.03 | 0.06 | 20 | 3051493 | TSS1500 | N_Shore |
| cg04528380 | 1.31 | 0.20 | 0.21 | 20 | 3051528 | TSS1500 | N_Shore |
| cg04731988 | 1.87 | 0.07 | 0.10 | 20 | 3051954 | TSS1500 | N_Shore |
| cg19776589 | 2.08 | 0.04 | 0.07 | 20 | 3052058 | TSS1500 | N_Shore |
| cg07747220 | 2.31 | 0.03 | 0.06 | 20 | 3052115 | TSS200 | Island |
| cg16887334 | 2.42 | 0.02 | 0.06 | 20 | 3052151 | TSS200 | Island |
| cg13285174 | 2.73 | 0.009 | 0.06 | 20 | 3052221 | TSS200 | Island |
| cg26267561 | 2.58 | 0.01 | 0.06 | 20 | 3052224 | TSS200 | Island |
| cg01644611 | 1.78 | 0.08 | 0.11 | 20 | 3052253 | TSS200 | Island |
| cg13725599 | 2.34 | 0.02 | 0.06 | 20 | 3052262 | TSS200 | Island |
| cg26955850 | 1.69 | 0.10 | 0.12 | 20 | 3052345 | 1stExon | Island |
| cg09774842 | 2.23 | 0.03 | 0.06 | 20 | 3052483 | Body | Island |
| cg06404175 | 2.72 | 0.009 | 0.06 | 20 | 3052692 | Body | Island |
| cg12099952 | 1.46 | 0.15 | 0.17 | 20 | 3053037 | Body | Island |

The statistical threshold was set at corrected *FDR* < 0.05 with the Benjamini-Hochberg (BH) adjustment for multiple comparisons

**Supplementary Table S2.** Statistical analysis results using multiple regression for each CpG fragment

| CPG.Labels | *T*.statistic | *P*.value | *FDR* (BH) | CHR | MAPINFO | EPIC probe |
| --- | --- | --- | --- | --- | --- | --- |
| **CpG_3,4** | **2.79** | **0.008** | **0.04** | **20** | **3052098/3052100** | |
| CpG_5 | 2.47 | 0.02 | 0.06 | 20 | 3052115 | cg07747220 |
| CpG_6 | 1.29 | 0.20 | 0.26 | 20 | 3052147 |  |
| CpG_8 | 1.92 | 0.06 | 0.12 | 20 | 3052157 |  |
| **CpG_9,10** | **2.91** | **0.006** | **0.04** | **20** | **3052169/3052172** |  |
| CpG_13 | 2.17 | 0.03 | 0.09 | 20 | 3052253 | cg01644611 |
| CpG_14,17 | 0.90 | 0.37 | 0.41 | 20 | 3052262/3052296 | cg13725599/ |
| CpG_16 | 1.70 | 0.10 | 0.16 | 20 | 3052290 |  |
| CpG_18 | -0.05 | 0.96 | 0.96 | 20 | 3052307 | |
| CpG_22 | 1.34 | 0.19 | 0.26 | 20 | 3052355 |  |

The statistical threshold was set at corrected *FDR* < 0.05 with the Benjamini-Hochberg (BH) adjustment for multiple comparisons

**Supplementary Figure S1.** Correlation between EPIC probes (cg07747220, cg01644611, and partially cg13725599) and EpiTYPER fragments (CpG 5, 13, and 14). CpG14, 17 was an averaged methylation from both the sites, and we were not able to separate CpG14 from CpG17 by EpiTYPER due to a technical limitation.


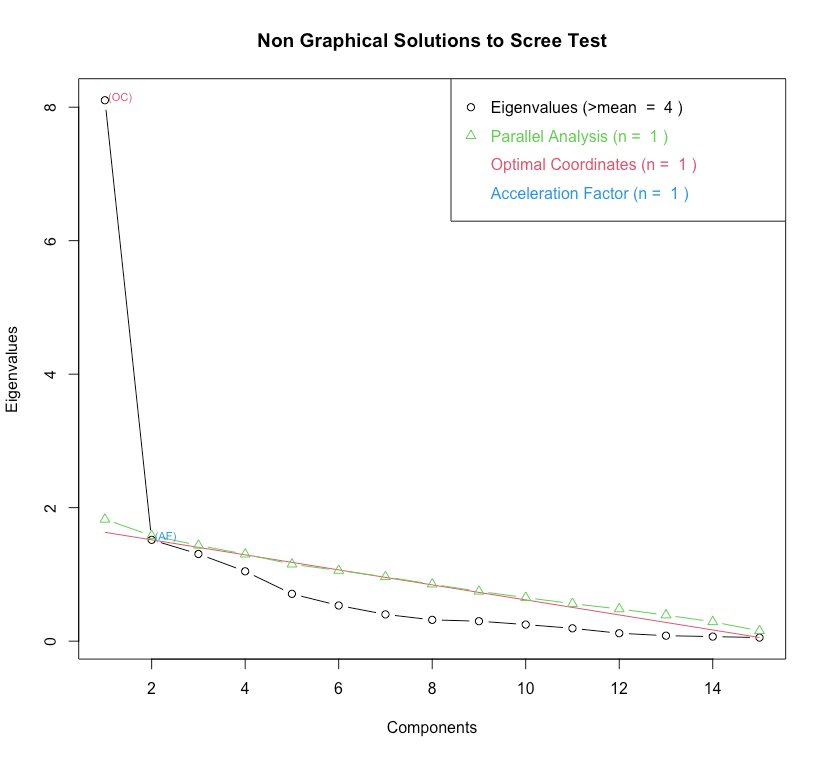


**Supplementary Figure S2.** Scree plot for the eigenvalues.

**Supplementary Table S3.** Maximum likelihood factor analysis (MLFA) using varimax rotation for EPIC probes

|  | **Factor1** | Factor2 | Factor3 | Factor4 |
| --- | --- | --- | --- | --- |
| cg09129163 |  |  |  |  |
| cg07597882 |  | 0.622 |  |  |
| cg04528380 |  | 0.682 |  |  |
| **cg04731988** | **0.606** |  |  |  |
| **cg19776589** | **0.822** |  |  |  |
| **cg07747220** | **0.727** |  |  |  |
| **cg16887334** | **0.874** |  |  |  |
| **cg13285174** | **0.777** |  |  |  |
| **cg26267561** | **0.606** | 0.567 |  |  |
| **cg01644611** | **0.844** |  |  |  |
| **cg13725599** | **0.881** |  |  |  |
| **cg26955850** | **0.679** |  |  |  |
| cg09774842 |  |  | 0.702 |  |
| cg06404175 |  |  |  |  |
| cg12099952 |  | 0.538 |  |  |

Factor 1 (Eigenvalue 8.15, loadings 0.606–0.881, cutoff = 0.500) that explained 36.6% of the variance.

**Supplementary Figure S3.** Correlation matrices between each pair of (A) fifteen EPIC probe methylation and (B) ten EpiTYPER fragments methylation. The size and color of the circles reflect correlation coefficients for each pair of the Pearson’s correlation analysis.

**Supplementary Table S4.** Background of CM subgroups

|  | PA+ (*n* = 16) | PA- (*n* = 8) | Statistics | *P* |
| --- | --- | --- | --- | --- |
| Male participants, *n* (%) | 13 (81.3) | 3 (37.5) | *χ^2^*(1) = 4.1 | 0.04 |
| Age (years), *Mean* (*SD*) | 12.7 (1.8) | 13.9 (3.0) | *t*(22) = -1.2 | 0.26 |
| Types of maltreatment, *n* (%)  Physical abuse  Emotional abuse  Neglect  Sexual abuse | 16 (100.0)  16 (100.0)  14 (87.5)  2 (12.5) | 0 (0.0)  2 (25.0)  7 (87.5)  0 (0.0) | NA  NA  *χ^2^*(1) = 0  NA | NA  NA  1  NA |
| #Types of maltreatment | 3.0 (0.5) | 1.1 (0.4) | *t*(22) = 9.2 | 5.4E-09 |
| Perpetrators, *n* (%)  Biological mother / father  Others | 13 (81.3) / 9 (56.3)  7 (43.8) | 5 (62.5) / 7 (87.5)  0 (0.0) | *χ^2^*(1) = 1.0 / *χ^2^*(1) = 2.3  NA | 0.32 / 0.13  NA |
| Duration of maltreatment |  |  |  |  |
| Years / %years of their lives | 9.1 (3.6) / 71.4 (26.0) | 3.9 (1.8) / 30.6 (19.4) | *t*(22) = 3.8 / *t*(22) = 3.9 | 8.8E-04 / 7.9E-04 |
| Duration elapsed from maltreatment |  |  |  |  |
| Years / %years of their lives | 3.5 (3.2) / 27.8 (25.6) | 10.0 (4.8) / 68.7 (25.6) | *t*(22) = -4.0 / *t*(22) = -3.7 | 6.4E-04 / 1.3E-03 |
| WISC-IV (FSIQ) | 87.7 (10.5) | 91.6 (10.9) | *t*(22) = -0.9 | 0.40 |
| CATS total | 41.8 (21.0) | 20.4 (11.5) | *t*(20) = 2.5 | 0.02 |
| SDQ total | 12.1 (8.1) | 9.6 (6.3) | *t*(22) = 0.8 | 0.45 |
| CBCL total | 61.8 (8.1) | 58.9 (8.0) | *t*(22) = 0.8 | 0.41 |
| DSRSC | 13.1 (7.4) | 11.5 (5.1) | *t*(21) = 0.6 | 0.59 |
| IWMS |  |  |  |  |
| Secure | 20.5 (7.8) | 22.1 (4.6) | *t*(20) = -0.5 | 0.61 |
| Avoidant | 14.9 (5.0) | 12.4 (3.8) | *t*(20) = 1.1 | 0.27 |
| Ambivalent | 16.3 (7.1) | 16.9 (3.4) | *t*(20) = -0.2 | 0.86 |
| Insecure | 31.2 (11.2) | 29.3 (6.4) | *t*(20) = 0.4 | 0.68 |

**Supplementary Figure S4.** Comparison of the *OXT*mi between PA+, PA-, and non-CM. Left: exact plot reflected the statistical result of the partial correlation analysis adjusted for age, gender, FSIQ, and proportion of buccal epithelium cells. Right: semi-partial plot using unadjusted “Group” for visually better understanding.

**Supplementary Figure S5.** Gene expression of *OXT* in the developing human brain. The figure shows the *OXT* gene expression measured via RNA-seq in BrainSpan (http://www.brainspan.org) datasets (*n* = 15; mean age: 11.9 ± 8.9; age range: 1-30 years). Dashed line represents mean expression level across all the regions (0.13). Error bars represent standard errors of the mean.

**Supplementary Table S5.** *OXT* methylation correlations between brain and saliva from the IMAGE-CpG database (Braun PR et al., 2019)

|  |  | **EPIC** | | **450K** | |
| --- | --- | --- | --- | --- | --- |
| **Probe ID** | **Chr:MAPINFO** | ***rho*** | ***P*** | ***rho*** | ***P*** |
| cg09129163 | Chr20:3050786 | -0.12 | 0.60 | NA | NA |
| cg07597882 | Chr20:3051493 | -0.34 | 0.13 | NA | NA |
| cg04528380 | Chr20:3051528 | -0.21 | 0.36 | NA | NA |
| cg04731988 | Chr20:3051954 | -0.43 | 0.05 | 0.16 | 0.66 |
| **cg19776589** | Chr20:3052058 | -0.20 | 0.39 | -0.10 | 0.79 |
| **cg07747220** | Chr20:3052115 | -0.44 | 0.05 | -0.22 | 0.54 |
| **cg16887334** | Chr20:3052151 | -0.19 | 0.42 | 0.08 | 0.84 |
| **cg13285174** | Chr20:3052221 | -0.10 | 0.67 | 0.03 | 0.95 |
| **cg26267561** | Chr20:3052224 | -0.21 | 0.36 | 0.10 | 0.79 |
| **cg01644611** | Chr20:3052253 | -0.12 | 0.59 | -0.03 | 0.95 |
| **cg13725599** | Chr20:3052262 | -0.05 | 0.83 | -0.05 | 0.89 |
| cg19592472* | Chr20:3052274 | -0.40 | 0.07 | 0.15 | 0.68 |
| **cg26955850** | Chr20:3052345 | 0.07 | 0.77 | 0.84 | 0.00 |
| cg09774842 | Chr20:3052483 | 0.14 | 0.53 | 0.47 | 0.18 |
| cg06404175 | Chr20:3052692 | 0.54 | 0.01 | 0.28 | 0.43 |
| cg12099952 | Chr20:3053037 | 0.10 | 0.65 | 0.16 | 0.66 |

**Bold**: probes constructed *OXT*mi

*: removed by quality control process

**Supplementary Table S6.** Numbers of participants in each brain imaging data

| *N* | T1 | DTI | fMRI | rs-fMRI |
| --- | --- | --- | --- | --- |
| CM | 24 | 22 | 14 | 11 (PA+: 7, PA-: 4) |
| non-CM | 31 | 31 | 19 | 0 |
| Total | 55 | 53 | 33 | 11 |
