# Supplementary material for: A multi-modal MRI analysis of brain structure and function in relation to OXT methylation in maltreated children and adolescents
Source: Transl Psychiatry. 2021 Nov 18;11:589. doi: 10.1038/s41398-021-01714-y (PMC8599663; doi:10.1038/s41398-021-01714-y)
Supplement: Supplementary file 2 — Supplementary Methods [file 41398_2021_1714_MOESM2_ESM.docx]

**Supplementary methods**

Brain image acquisition and preprocessing

Image acquisition was carried out using a 3-Tesla scanner (Discovery MR 750; General Electric Medical Systems, Milwaukee, WI, USA) with a 32-channel head coil. Functional images were acquired with a T2*-weighted gradient-echo echoplanar imaging (EPI) sequence. For functional imaging using monetary reward tasks, a series of 528 volumes (132 volumes per session) was acquired and each volume consisted of 44 transaxial slices with a thickness of 3.0 mm between slices (repetition time (TR)= 3,000 ms; echo time (TE)=25 ms; flip angle (FA)=90°; field of view (FOV)=192 mm; in-plane matrix size=64 × 64 pixels, voxel dimensions=3.0 × 3.0 × 3.0 mm; slice gap=0 mm). Resting-state functional images were acquired; each volume consisted of 40 slices, with a thickness of 3.5 mm and a 0.5-mm gap (TR=2,300 ms; TE=30 ms; FA=81°; FOV=192×192 mm; 64×64 matrix; yielding volume dimensions of 3.0×3.0 mm). A total of 192 volumes were acquired for an imaging time of 7 min 42 s. The participants were instructed to remain awake but to close their eyes and think of nothing in particular. Head movement was minimized by the placement of memory-foam pillows around the head. For structural imaging, High-resolution images were acquired by a 3D T1-weighted fast spoiled gradient recalled imaging sequence (TR=6.38 ms; TE=1.99 ms; FA=11°; FOV=256 mm; 256×256 matrix; 172 slices; voxel dimension =1.0×1.0×1.0 mm). For DTI imaging, a single-shot, spin-echo, EPI sequence was used to acquire 64 axial planes with 30 non-collinear diffusion sensitization gradients (b=1,000 s/mm^2^), and a reference image with no diffusion weighting (b0 image) (TR=8,400 ms, TE=84.2 ms, FOV=256×256 mm^2^, matrix size = 256 × 256, slice thickness = 2 mm with no gap).

Brain image preprocessing

The functional imaging data were preprocessed using Statistical Parametric Mapping (SPM) 12 (Wellcome Trust Centre for Neuroimaging, London, UK) with MATLAB R2016b (MathWorks, Natick, MA, USA). First, the first four volumes of each fMRI session were discarded to allow for stabilization of the magnetization, and the remaining 128 volumes were used for analysis. Following realignment, all images were normalized to the SPM12 image template. The anatomically normalized EPI data were spatially smoothed in three dimensions using an 8-mm full-width half-maximum Gaussian kernel.

Resting-state functional data were preprocessed using the CONN toolbox in SPM12. A default preprocessing pipeline for the volume-based analyses was conducted. Before the pre-processing, we removed the initial 4 volumes to allow for image intensity stabilization. In this process, images were realigned, unwarped, and slice-time corrected. Then, the images were simultaneously segmented into gray matter, white matter, and CSF, and normalized to the standard MNI space. In a final step, the images were spatially smoothed using an 8 mm FWHM Gaussian kernel. Finally, denoising was performed.

The structural imaging data were also preprocessed using VBM implemented in SPM12. The T1-weighted images were segmented coarsely into gray matter (GM), white matter (WM), cerebrospinal fluid, and skull/scalp compartments using tissue probability maps. The Diffeomorphic Anatomical Registration through Exponentiated Lie Algebra (DARTEL) algorithm was applied to the segmented brain tissues to generate a study-specific template and to achieve an accurate inter-subject registration with improved realignment of smaller inner structures. The segmented GM images were spatially normalized with an isotropic voxel resolution of 1.5 mm. Any volume change induced by normalization was adjusted using a modulation algorithm. Spatially normalized GM images were smoothed using a Gaussian kernel with a full width at half maximum of 10 mm.

The DTI data were preprocessed using the Oxford Centre for Functional MRI of the Brain (FMRIB) Software Library (FSL; http://www.fmrib.ox.ac.uk/fsl). First, head movement and eddy current distortion were corrected and aligned to the reference image (b0 image). Next, the non-brain tissue was removed using the brain extraction tool. Finally, to generate individual FA maps for each participant, the diffusion tensor model was fitted to each voxel using FMRIB's Diffusion Toolbox.

Monetary reward task for fMRI

Briefly, participants were asked to choose one of the three cards by pressing a button. Each card was randomly assigned to Japanese Yen (JPY) 0, 30, or 60 (100 JPY is equivalent to 1 US Dollar). Three conditions of eight trials were performed (24 s). Unknown to the participants, the total reward was predetermined. Two reward conditions with different levels were designed to investigate the effects of reward levels between groups. In the high monetary reward (HMR) condition, participants earned an average of 330 JPY (range 270–390), which was consistently higher than the expected value of the eight reward trials (240 JPY). In the low monetary reward (LMR) condition, participants earned an average of 150 JPY (range, 90–210), which was consistently lower than the expected value. In the no monetary reward (NMR) condition, the outcome presented was always ‘XXX’ to control for effects other than reward level. The NMR condition or a fixation rest condition (24 s) was always inserted between the two reward conditions. During the scanning, the participants performed four sessions. Each session consisted of four blocks from each of the four conditions (HMR, LMR, NMR, and fixation rest). The order of the four sessions was balanced across participants.

Statistical analysis for DTI

We specifically focused on FA, a DTI-derived measure of WM organization. The TBSS tool in the FSL was used for the voxel-wise analysis of pre-processed FA data. First, individual FA images were aligned to the FMRIB58_FA template and transformed into the Montreal Neurological Institute standard space. Next, all aligned FA images were averaged to generate a mean FA image, which was then thinned to create a mean tract skeleton. The tract skeleton was thresholded at an FA value of ≥ 0.2 to exclude peripheral tracts and minimize the partial volume effect. Finally, each participant's aligned FA images were projected onto the mean tract skeleton, and the resulting data were fed into a voxel-wise permutation-based analysis. In addition, MD, AD, and RD can also be assessed from the elements of the diffusion tensor matrix, that is, the three eigenvalues λ1, λ2, and λ3. MD was defined as the mean of all three eigenvalues [(λ1±λ2±λ3)/3], AD as the principal diffusion eigenvalue (λ1), and RD as the mean of the second and third eigenvalues [(λ2±λ3)/2].
